# Access to and quality use of non-communicable diseases medicines in Nepal

**DOI:** 10.1186/s40545-015-0041-7

**Published:** 2015-08-31

**Authors:** Bhuvan K.C., Susan Heydon, Pauline Norris

**Affiliations:** School of Pharmacy University of Otago, 18 Frederick Street, Dunedin, 9054 New Zealand

## Abstract

Noncommunicable diseases are a major healthcare problem in Nepal and their burden is increasingevery year. Noncommunicable diseases (NCDs) bring additional challenges to the Nepalese healthcaresystem which is already experiencing infrastructure shortages, poor service delivery, inadequate essential medicines coverage and shortages of healthcare workers. The Nepal government provides a limited number of free essential medicines through the free essential healthcare services program. This consists of a basic healthcare package provided through primary healthcare (PHC) facilities and district hospitals. Though around 40 essential medicines are provided without charge, studies have reported problems with access especially in all rural areas. There is a need to improve access to, coverage and quality use of medicines. The government has decided to provide some free medicines for NCDs alongside free essential medicines to be distributed via current healthcare structures. Though well intended, this decision will put extra strain on the essential medicines program. It should be supplemented by a comprehensive NCDs policy that takes account of the issues of sustainability and quality use of medicines. Complex cases of NCDs will be managed by tertiary hospitals but most of the cases of NCDs especially for rural people and the poor will end up in secondary level public hospitals (district and zonal hospitals). Therefore, the government needs to strengthen these public hospitals. Meanwhile, given the severity of the NCDs problem in Nepal, the Ministry of Health and Population (MoHP) should liaise with nongovernmental and missionary hospitals especially in rural areas to run NCDs management services. The Ministry should encourage these hospitals to run hospital pharmacies to improve people’s access to and quality use of NCDs medicines. At the primary healthcare level, the Ministry could run NCDs prevention and control programs but existing PHC workers need training to perform proper dispensing of NCDs medicines. PHC facilities need a medical record system so that they can address the needs of NCDs patients requiring long term medication supply via a proximate PHC facility.

## Background

### The public healthcare system in Nepal

Access to essential medicines for non-communicable diseases (NCDs) is a concern for Nepal as the incidence of NCDs in Nepal is increasing. The Nepal Government has approved the Ministry of Health and Population’s (MoHP) decision to increase the number of free essential medicines from 40 to 70 by including medicines for NCDs, without developing comprehensive policy for NCDs [1]. This commentary analyses the impact of the decision on the quality use of medicines in Nepal and explores alternative mechanisms for NCDs medicines distribution.

Nepal is a low-income country in South Asia located between China and India. It has a population of 28.1 million, [2] of whom 44.2 % are living below the poverty line as determined by the Multidimensional Poverty Index [3]. Health service delivery in Nepal lacks quality and universal coverage. Public health services are delivered via tertiary hospitals, regional and zonal hospitals in urban areas and via district hospitals, primary healthcare centers, health posts and sub-health posts in rural areas [4]. The Nepal Government initiated the free essential healthcare services (EHCS) program in 2007 which has now been scaled up to district hospital level [5]. The free EHCS program includes free primary healthcare (PHC) services, basic secondary care services and a limited number of free essential medicines [5]. People have to pay for all other health services in both the public and private sectors, and a significant proportion of healthcare financing consists of out-of-pocket expenditure (for example for medicines, doctors’ visits, lab tests, transportation and hospital stays) [6]. While policy initiatives towards universal health coverage and health sector reforms have been taken in order to improve health system efficiency, equity and accessibility of healthcare for underprivileged groups, [7] initiatives to improve the quality of healthcare services to retain patients at public health facilities are still lacking.

### The state of the free essential medicines program

After the initiation of the free essential healthcare services program in 2007, the government has gradually increased its coverage so as to provide free basic healthcare and a limited number of essential medicines [8]. Providing free essential medicines is the first step, but there is much to do to improve coverage and quality use of essential medicines. Ensuring quality use of medicine in the Nepalese context requires: updating standard treatment guidelines, establishing pharmacy and therapeutic committees in hospitals to monitor quality use of medicines, conducting rational drug use training, updating essential medicines lists and strengthening health logistics provisions especially in PHC facilities [9, 10]. This needs more resources and time. As of 2014, under the free EHCS program a limited number of medicines (25 at sub-health post and health posts, 35 at primary health care centers and 40 at district hospitals) are provided through primary healthcare facilities and district hospitals [7]. However, the actual need for medicines is much higher. A study report submitted to the National Planning Commission in 2012 showed that health service users (*N* = 100) reported year round availability of essential medicines to be 16.6 % in health facilities from the Mountains, 57.1 % in the Hills and 52.2 % in the Terai (plains in the southern part of Nepal which borders with India and where more than 50 % of the population lives) [11]. Year round availability of essential medicines was reported by only 25 % of users in district hospitals, 40 % in primary health care centers and 36.6 % in health posts [11]. A review of the free essential healthcare services program reported that both the type and quantities of free essential medicines were insufficient for PHC facilities and district hospitals, expired medicines were present in health facilities and human resources were inadequate [12]. Thus, there is a need to improve logistics, human resource capabilities and coverage of the essential medicines program so as to improve access to and quality use of medicines to achieve the goals of the free essential healthcare services program.

### Non-communicable diseases burden in Nepal and quality use of medicine

As stated in the Department of Health Services (DoHS) annual report 2013–14, 86 % of new in-patient visits (*N* = 287,616) were the result of NCDs [13]. Two NCDs (other chronic obstructive pulmonary disease and essential (primary) hypertension) were among the top ten causes of in-patient mortality in public hospitals during 2013–14 [13]. The majority of NCDs in Nepal are cardiovascular diseases, injuries and neuropsychiatric conditions, cancers, chronic respiratory diseases and diabetes [13]. The population aged 65 years and older is projected to rise from 4.2 % in 2000 to 5.8 % in 2025 and therefore, the burden of NCDs is going to increase [14]. The government has developed a multi-sectoral action plan on the prevention and control of NCDs which aims to reduce preventable morbidity, avoidable disability and premature mortality due to NCDs [15]. Consequently, the government announced a plan for including NCDs medicines in the free essential medicines program in its budget for the fiscal year 2011–12 [16]. These medicines would be distributed through the present healthcare structure including PHC facilities [16]. Government initiatives for distributing NCDs medicines could be an opportunity to integrate NCDs medicines with the existing free essential medicines program and improve people’s access to and quality use of medicines.

NCDs treatment requires long-term care enabled via a reliable healthcare facility and medicines supply system [17]. Experiences from Uganda and Rwanda show that chronic shortage of health workers, lack of access to basic health supplies and limited or unreliable financial resources were systemic challenges [18]. These are similar to Nepal in many ways. The NCDs prevention and control plan of Nepal included health system strengthening, especially PHC, which includes preparing a package of essential medicines for NCDs, improving the competency of PHC workers to handle NCDs management and the promulgation of a three tier system viz. first tier- PHC level, middle tier- zonal and district hospital level, third tier-speciality centres [15]. Therefore, the government should develop a comprehensive NCDs treatment policy with appropriate treatment guidelines for various NCDs and clear demarcation of roles of each level of health facilities in NCDs management.

Zonal and district hospitals are proposed as second line facilities for the treatment and management of NCDs in Nepal [15]. However, many public hospitals in Nepal especially in far-western, mid-western region and the Terai region are in poor condition [19]. These hospitals lack human resources (specialist doctors and paramedics), appropriate infrastructure and the financial resources required to perform NCDs management services [19] [20]. Most need upgrading, which is beyond the MoHP’s capacity. Nevertheless, some combined initiatives of the government and aid agencies to provide environmental support for rural staff have increased patient utilization in Bajhang, Gulmi and Dolakha districts [21]. Likewise, nongovernmental organization (NGOs) and mission hospitals have provided much needed secondary level care in Dadeldhura, Okhaldhunga, Lamjung, Palpa, Kavre and Accham districts [22]. Nepal today has enough pharmacists to cover all district hospitals to promote quality use of medicines [23, 24]. The MoHP thus needs to liaise with NGOs and mission hospitals to run NCDs treatment program in districts with limited public hospital serves and encourage district hospital management committees’ to develop hospital pharmacy so as to improve people’s access to and quality use of NCDs medicines.

Since distributing NCDs medicines through the free essential medicines program involves PHC facilities, they need to be strengthened. Nepal has a successful track record of employing primary healthcare workers such as auxiliary health workers, village health workers and auxiliary nurse midwives in immunization program, mass medication of albendazole and diethylcarbazide, distribution of anti-TB medicines, reproductive and maternal health programs [25, 26]. A systematic review from low-and-middle income countries also supports their use as an effective and affordable approach for making healthcare accessible for NCDs but such step needs to be accompanied by health system re-structuring [27]. Community health workers were also involved in the management of non-communicable health problem at community level in countries like Pakistan, India and Bangladesh [28]. The government needs to introduce training programs for these PHC workers so that they can distribute NCDs medicines without compromising quality use and establish a networking system between PHC facilities and public hospitals so that NCDs patients can be tracked and enrolled in NCDs treatment programs.

The government has proposed to distribute NCDs medicines by including them in the free essential medicines list and delivering them via the same healthcare structures [1] which will increase the logistics burden of the program. Furthermore, need for NCDs medicines will depend on the local prevalence data. Therefore, the government has to improve the health logistics infrastructure at regional and district level so that they can deliver needed NCDs medicines. Furthermore, upgrading medicines stores at district health offices and PHC facilities is required so that medicines can be stored properly and quality of medicines maintained.

## Conclusion

The distribution of NCDs medicines is a major and long term issue for which the government needs to ensure sustainability and quality use of medicines. The government needs to work towards prevention, control and management of NCDs. Complicated cases of NCDs will be managed at tertiary level but the majority of the NCDs patients will be treated in public hospitals at district and zonal level. The government needs to gradually strengthen these hospitals with appropriate human resources and infrastructure. However, given the seriousness of NCDs problem utilizing nongovernmental and mission hospitals at district level and encouraging them to run hospital pharmacies might improve access to and quality use of NCDs medicines. At PHC level, the focus should be on running comprehensive NCDs prevention and control programs and training existing PHC workers in rational dispensing of NCDs medicines. Such activity requires a comprehensive approach, sustainable financing and integration with a multi-donor program like Nepal Health Sector Support Programme.

## Abbreviations

DoHS: Department of Health Services
EHCS: Essential Health Care Services
GoN: Government of Nepal
MoHP: Ministry of Health and Population, Nepal
NCDs: Non-communicable diseases
PHC: Primary Health Care
TB: Tuberculosis
